# Comparison of TOF-PET and Bremsstrahlung SPECT Images of Yttrium-90: A Monte Carlo Simulation Study

**DOI:** 10.22038/aojnmb.2017.9673

**Published:** 2018

**Authors:** Akihiko Takahashi, Kazuhiko Himuro, Shingo Baba, Yasuo Yamashita, Masayuki Sasaki

**Affiliations:** 1 Division of Medical Quantum Science, Department of Health Sciences, Kyushu University, Fukuoka, Japan; 2 Division of Radiology, Department of Medical Technology, Kyushu University Hospital, Fukuoka, Japan; 3 Department of Clinical Radiology, Kyushu University Hospital, Fukuoka, Japan

**Keywords:** Bremsstrahlung SPECT, Monte Carlo simulation, TOF-PET, ^90^Y

## Abstract

**Objective(s):**

Yttrium-90 (^90^Y) is a beta particle nuclide used in targeted radionuclide therapy which is available to both single-photon emission computed tomography (SPECT) and time-of-flight (TOF) positron emission tomography (PET) imaging. The purpose of this study was to assess the image quality of PET and Bremsstrahlung SPECT by simulating PET and SPECT images of ^90^Y using Monte Carlo simulation codes under the same conditions and to compare them.

**Methods:**

In-house Monte Carlo codes, MCEP-PET and MCEP-SPECT, were employed to simulate images. The phantom was a torso-shaped phantom containing six hot spheres of various sizes. The background concentrations of ^90^Y were set to 50, 100, 150, and 200 kBq/mL, and the concentrations of the hot spheres were 10, 20, and 40 times of those of the background concentrations. The acquisition time was set to 30 min, and the simulated sinogram data were reconstructed using the ordered subset expectation maximization method. The contrast recovery coefficient (CRC) and contrast-to-noise ratio (CNR) were employed to evaluate the image qualities.

**Results:**

The CRC values of SPECT images were less than 40%, while those of PET images were more than 40% when the hot sphere was larger than 20 mm in diameter. The CNR values of PET images of hot spheres of diameter smaller than 20 mm were larger than those of SPECT images. The CNR values mostly exceeded 4, which is a criterion to evaluate the discernibility of hot areas. In the case of SPECT, hot spheres of diameter smaller than 20 mm were not discernable. On the contrary, the CNR values of PET images decreased to the level of SPECT, in the case of low concentration.

**Conclusion:**

In almost all the cases examined in this investigation, the quantitative indexes of TOF-PET ^90^Y images were better than those of Bremsstrahlung SPECT images. However, the superiority of PET image became critical in the case of low activity concentrations.

## Introduction

Yittrium-90 (^90^Y) is a beta particle radionuclide used for targeted radionuclide therapy in relapsing follicular lymphomas (1–4). In standard ^90^Y-ibritumomab treatments, pre-therapy estimation to predict ^90^Y accumulation is performed using indium-111(^111^In)-labeled ibritumomab (5). SPECT using the Bremsstrahlung gamma ray of the beta particle has been developed to detect ^90^Y directly (5–13); however, the gamma ray of ^111^In (171 keV, 245 keV photo-peaks, and Compton-scattered gamma rays) may contaminate the energy window of ^90^Y Bremsstrahlung single-photon emission computed tomography (SPECT). We investigated the crosstalk of ^111^In gamma ray and ^90^Y Bremsstrahlung in the SPECT imaging using in-house Monte Carlo simulation codes (14) and found that the count ratio of ^90^Y Bremsstrahlung was only less than 30% of the total counts (including ^111^In gamma ray) under probable conditions. ^111^In is an obstacle to direct detection of ^90^Y.

Positron emission tomography (PET) imaging is a powerful alternative to SPECT. ^90^Y emits positrons due to internal pair production; therefore, PET imaging, which is not affected by the gamma ray of ^111^In, is possible (15–24). However, the branching ratio of internal pair production is very small (~3.2×10^−5^) (25). Therefore, it is important to quantitatively compare PET and Bremsstrahlung SPECT (23, 24). Elschot et al. conducted systematic experiments of ^90^Y PET and SPECT imaging under considerably high background activity concentration (0.27–1.09 MBq/mL) conditions and concluded that the image quality of PET is superior to that of Bremsstrahlung SPECT (24).

We also investigated ^90^Y PET and SPECT imaging using a Monte Carlo simulation (26), in which the kinetics and the image qualities of both the imaging processes were compared. Our results showed that the detection threshold and the background noise level of PET images are lower than those of SPECT. As a result, the image quality of PET images seemed to be superior to that of SPECT. However, the background activity was not set to specific conditions in this simulation; thus, we cannot conclude the superiority of PET to Bremsstrahlung SPECT in realistic conditions.

The aim of this study was to assess the image quality of PET and Bremsstrahlung SPECT for more realistic settings and to compare them. In this study, we performed simulations of ^90^Y PET and Bremsstrahlung SPECT imaging with background activities of 50–200 kBq/mL under the same hot-to-background ratio and acquisition time. The simulated images were evaluated by two quantitative indexes: contrast recovery coefficient (CRC) and contrast-to-noise ratio (CNR).

## Methods

### Phantom

**Figure 1:**
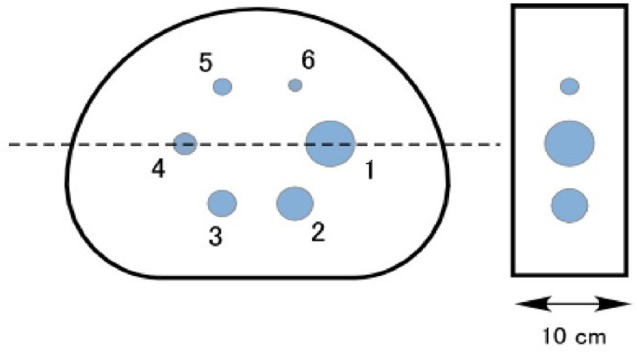
Cross-sectional and side views of phantom. Inner diameters of spheres: 37, 28, 22, 17, 13, and 10 mm.

Figure 1 shows the cross-section and side view of the phantom. The National Electrical Manufacturers Association (NEMA) 2007/IEC 2008 PET image quality phantom was used for all the simulations (27). The phantom comprises a fillable torso-shaped compartment containing six fillable coplanar spheres (inner diameters: 37, 28, 22, 17, 13, and 10 mm). The axial length was set at 10 cm to save the active volume (5400 mL). The numbers in the figure are the number of hot sphere regions of interest (ROIs), and the sizes of these ROIs were the same as the hot spheres. The background activity concentration was set at 200, 150, 100, and 50 kBq/mL, and the activity concentration in the spheres was 40, 20, and 10 times of each background concentration, based on previous studies (20, 22, 24). The acquisition time was 30 min in both PET and SPECT.

### Simulation Codes

In-house Monte Carlo simulation codes “MCEP-PET” and “MCEP-SPECT,” previously reported in detail (26) and based on the simulation code developed by Uehara et al. (28, 29) were used for this study. The MECP-PET produces a number of detected photons and then builds up the sinogram. The sinogram was reconstructed using a time-of-flight ordered subset expectation maximization (TOF-OSEM) algorithm. We optimized the conditions by checking the change in the pixel values inside the region of interest for every iteration. Table 1 lists the basic specifications for PET scanner and image reconstruction. The reconstruction process included attenuation correction; the reconstructed image was filtered by a Gaussian function filter with a 6-mm full width at half maximum (FWHM).

**Table 1:** Basic specifications for MCEP-PET

Crystal material	LSO
Crystal size	4 ×4×20 mm^3^
Number of crystals/ring	624
Ring diameter	842 mm
Number of rings	39
Transversal FOV (field of view)	600 mm
Axial FOV	162 mm
Coincidence timing window	4.5 ns
Energy window	435–650 keV
TOF resolution	550 ps
Acquisition time	30 min
Acquisition mode	3D
Image matrix size	312×312
Pixel size	1.9 mm
OSEM subset × iteration	8×3
FWHM of Gaussian filter	6 mm

The background due to ^176^Lu decay in LSO crystals was effectively removed by the sophisticated random correction in the state-of-the-art PET; therefore, we curtailed the ^176^Lu decay in this simulation. We tried random correction by the delay coincidence method in this study; however, the results were hardly influenced under the conditions in this study. We also tried the scatter correction by raising the lower energy limit of energy window from 435 kev to 500 keV. Thus, the CNR value slightly decreased to a concentration lower than 100 kBq/mL because of the lower count number. Therefore, we adopted the results without scatter correction. Recently, Strydhorst et al. reported a Monte Carlo simulation of ^90^Y in PET using the GATE simulation code (30). They concluded that the scatter, ^90^Y bremsstrahlung, and the LSO background all *slightly* degrade the observed contrast ratio.

MCEP-SPECT simulates a dual-detector system and produces projection images (26). The set of projection images were reconstructed using the OSEM algorithm in the imaging software package for nuclear medicine-the Prominence Processor (version 3.1, distributed by the Prominence Conference, not for sale). Regarding SPECT images, sophisticated reconstruction methods have been developed and reported (31, 32). These methods are certainly promising and academically interesting, but not so easy to apply at present; therefore, we thought that an ordinary method is practical for the aim of this study. Table 2 lists the basic specifications for SPECT and image reconstruction. The projection images were pre-processed by a Butterworth filter (eighth-order, cut-off frequency: 0.5 cm^−1^) and reconstructed with attenuation correction (Chang method).

**Table 2:** Basic specifications for MCEP-SPECT

Radius of rotation of camera	260 mm
Energy window	105–195 keV
Number of projections	120 projections/360
Acquisition time	30 min
Collimator	HEGP
NaI crystal size	400×400×9.4 mm^3^
Matrix size	256×256
Pixel size	1.6×1.6 mm^2^
OSEM subset × iteration	8×8
Cut-off frequency of Butterworth filter	0.5 cycle/cm
Order of Butterworth filter	8

### Image analysis

The simulated ^90^Y images were quantitatively evaluated using the CRC, *Q*_H_ and the CNR, *ν*_H_.

Here, subscript *j* is the number of the hot sphere (see Figure 1), *C*_H,*j*_ is the mean activity concentration in the *j*-th hot sphere ROI, *C*_B_ is the mean activity concentration in the background ROI, *R* is the true hot-to-background activity concentration ratio (40, 20, and 10), and *SD*_B_ is the standard deviation of the activity concentration in the background ROI.


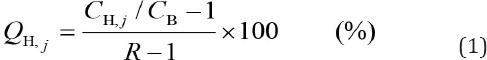



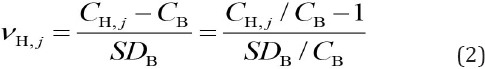


CRC is the percentage of measured net concentration normalized by the measured background concentration to true net concentration normalized by true background concentration. In other words, CRC indicates the accuracy of measurement. CNR is the ratio of net concentration to background fluctuation that might be false positives. Therefore, CNR indicates the detectability of the hot area. It states that an object is discernable when CNR is more than 4 (the Rose criterion) (33). CRC and CNR depend on the background ROI. In this investigation, we simulated the images without hot spheres and obtained *C*_B_ and *SD*_B_ using the whole cross-section of the central slice.

## Results

**Figure 2:**
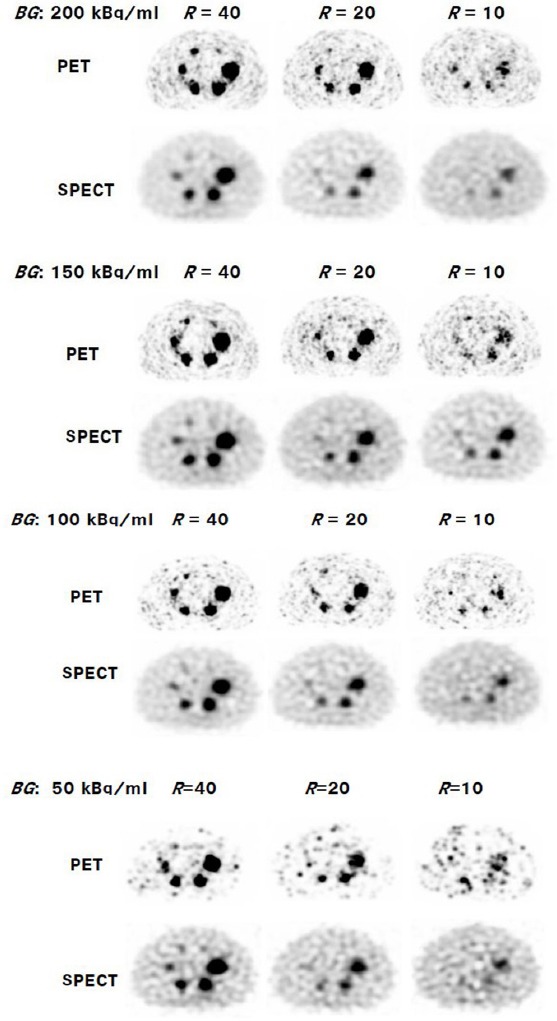
Simulated reconstructed images using ^90^Y-PET and SPECT. *BG* denotes the background activity concentration, and *R* denotes the hot-to-background ratio.

Figure 2 shows the simulated ^90^Y PET and SPECT images. In general, smaller hot spheres become discernable as the hot-to-background ratio (*R*) increases. When *R* was 40, hot spheres of diameter more than 13 mm were discernable in all cases. When the background activity concentration (*BG*) was more than 150 kBq/mL and *R* was 40 in PET images, hot spheres of 10 mm were discernable. On the contrary, sharpened PET images look rough; therefore, hot spots that might be false positives increased as the activity concentration decreased. As the result, the superiority of PET images visually became critical.

**Figure 3:**
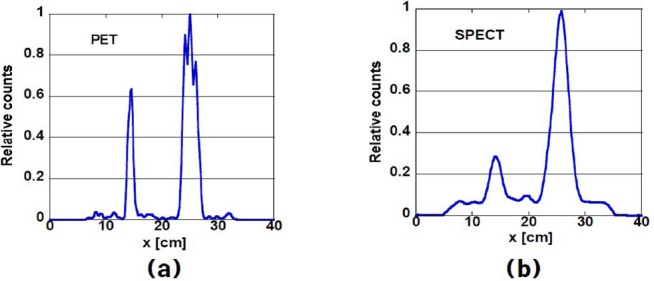
Activity concentration profiles on the horizontal dashed line shown in Figure 1 of (a) PET and (b) SPECT image. The background concentration was 200 kBq/mL, and the hot-to-background ratio was 40.

Figure 3 shows the simulated activity concentration profiles on the horizontal dashed line of the phantom shown in Figure 1. The background concentration was 200 kBq/mL, and the hot-to-background ratio was 40. The partial volume affects the SPECT image more strongly than the PET image and will, therefore, affect the CRC and CNR values.

**Figure 4:**
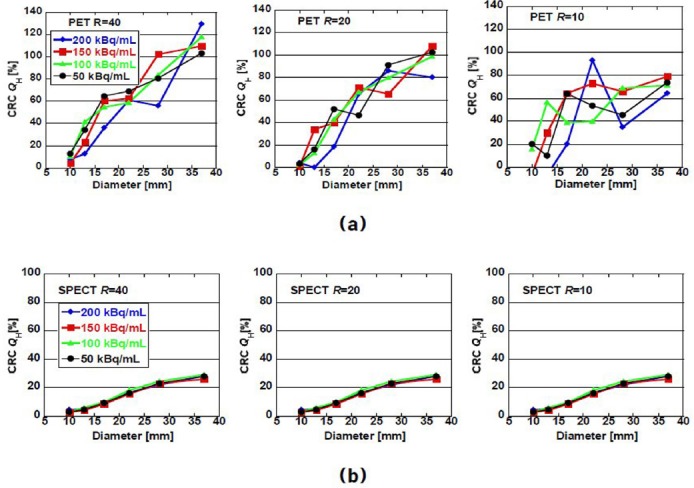
Constant recovery coefficient *Q*_H_ of (a) PET and (b) SPECT images as a function of sphere diameter for background activity concentration of 200, 150, 100, and 50 kBq/mL. *R* denotes the hot-to-background ratio.

**Figure 5:**
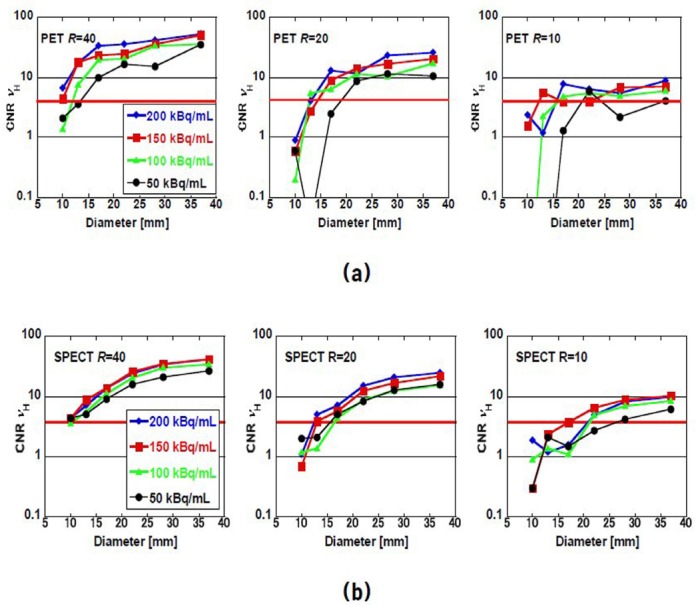
Constant-to-noise ratio *ν_H_* of (a) PET and (b) SPECT images as a function of sphere diameter for background activity concentration of 200, 150, 100, and 50 kBq/mL. *R* denotes the hot-to-background ratio. Horizontal solid line at *ν_H_* = 4 is the Rose criterion.

Figures 4 and 5 show the CRCs and CNRs of the images shown in Figure 2 as a function of the diameter of the hot sphere, respectively. In general, CRC increased as the size of the hot sphere and/or *R* increased. In the case of SPECT images, CRC did not exceed 40%. The dependence on the size of the hot sphere did not change much with the activity concentration and *R*. In the case of PET images, CRC exceeded 40% for the diameters more than 22 mm. The value depended on *R* more strongly than in the case of SPECT images. In a few cases, when the background concentration was below 100 kBq/mL, the CRC exceeded 100%. This is because the reconstructed image partially overshoots due to low detected counts.

CNR, which is the index of detectability of hot area, also increased as the size of the hot sphere and/or *R* increased. In the case of PET images, for background concentrations above 100 kBq/mL, CNR of spheres larger than 22 mm exceed the Rose criterion (*ν_H_*= 4), which means the spheres were discernable. The criterion seems to be consistent with the visual impression of the images shown in Figure 2. When *R* was 40, all spheres including the sphere of 10-mm diameter were discernable. In the case of SPECT, the threshold of the sphere size was larger than that of PET. However, the superiority of PET decreased when the activity concentration lowered. Specifically, the background concentration was 50–100 kBq/mL and *R* was 10; some CNRs of PET were smaller than those of SPECT.

## Discussion

The purpose of this study was to assess the superiority of ^90^Y PET images over Bremsstrahlung SPECT images. The acquisition time (30 min), background activity concentration *BG* (50–200 kBq/mL), and hot-to-background concentration ratio *R* (10–40) were common in both cases. The CRC of PET images were larger than those of SPECT under these conditions. The CNR of PET was also larger than those of SPECT. However, the superiority became critical in cases of low concentration (<100 kBq/mL) and/or small size (<20 mm) of the hot sphere.

It is obvious that the quality of PET images is superior to the SPECT ones for *R* above 20. The CRC values, which indicate the accuracy of measurement, of PET images were larger than those of SPECT in almost all cases. This is due to the higher sensitivity of time-of-flight positron emission tomography (TOF-PET) images. In contrast, the good sensitivity made the visual impression of PET images somewhat rough because the adventitious decay spots are effectively counted. The roughness becomes significant particularly in lower activity concentrations because of statistical fluctuation. This causes the increase of *SD*_B_ /*C*_B_ in equation (2) and depresses the CNR value.

The increase in roughness visually and quantitatively degraded the detectability of the hot area. Visually, hot spheres smaller than 20 mm became indiscernible when the *R* was less than 20 and the *BG* was less than 100 kBq/mL in both PET and SPECT images. The CNRs shown in Figure 5 qualitatively proved the visual impression. It should be noted that the CNRs of PET images rapidly degraded for *R* below 20 and *BG* below100 kBq/mL and became smaller than SPECT in contrast to the results for other cases. This is due to the increase of *SD*_B_ /*C*_B_ in equation (2) for lower activity concentration.

In our previous study, where we investigated the crosstalk between ^111^In and ^90^Y SPECT, the *BG* and the *R* were set to 39 kBq/mL and 10, respectively, as a clinically probable condition (14). According to the present assessment, the superiority of PET images to SPECT ones is somewhat critical for this condition. However, it is worth choosing PET for direct imaging of ^90^Y, considering that it was better at detecting lesions larger than 20 mm than SPECT and the serious crosstalk of pre-therapy ^111^In can be removed.

The limitation of this study is the reconstruction algorithm. The PET image quality depends on the merit of the scanners and reconstruction algorithm (20, 22). We used the in-house hand-made OSEM code, which might be inferior compared to the sophisticated software that equips state-of-the-art PET scanners; therefore, the reconstructed images might also be somewhat inferior to the real clinical images, especially for lower concentrations. For example, the overestimated CRC values in 100, 50 kBq/mL might be due to our unsophisticated algorithm.

## Conclusion

In this study, we quantitatively compared ^90^Y PET images reconstructed by TOF-OSEM and ^90^Y Bremsstrahlung SPECT images reconstructed by OSEM using Monte Carlo simulation codes. The quantitative indexes of TOF-PET ^90^Y images were better than those of Bremsstrahlung SPECT images. However, the superiority of PET images became critical as the activity concentration lowered to a value of 100 kBq/mL.
